# High cancer mortality for US-born Latinos: evidence from California and Texas

**DOI:** 10.1186/s12885-017-3469-0

**Published:** 2017-07-11

**Authors:** Paulo S. Pinheiro, Karen E. Callahan, Scarlett Lin Gomez, Rafael Marcos-Gragera, Taylor R. Cobb, Aina Roca-Barcelo, Amelie G. Ramirez

**Affiliations:** 1 School of Community Health Sciences, University of Nevada Las Vegas, 4505 S. Maryland Pkwy, Las Vegas, NV 89154 USA; 2 Cancer Prevention Institute of California, 2201 Walnut Ave., Suite 300, Fremont, CA 94538 USA; 3 Epidemiology Unit and Girona Cancer Registry, Catalan Institute of Oncology, Biomedical Research Institute (IdiBGi), Carrer del Sol 15, 17004 Girona, Spain; 4 Department of Epidemiology and Biostatics, Institute for Health Promotion Research, University of Texas Health Science Center at San Antonio, 411 John Smith, Suite 1000, San Antonio, TX 78229 USA

**Keywords:** Cancer, Hispanics, Latinos, Mortality, Nativity, Birthplace, Mexican, Texas, California, Immigrants

## Abstract

**Background:**

Latinos born in the US, 36 million, comprise 65% of all US Latinos. Yet their cancer experience is nearly always analyzed together with their foreign-born counterparts, 19 million, who constitute a steady influx of truly lower-risk populations from abroad. To highlight specific cancer vulnerabilities for US-born Latinos, we compare their cancer mortality to the majority non-Latino white (NLW) population, foreign-born Latinos, and non-Latino blacks.

**Methods:**

We analyzed 465,751 cancer deaths from 2008 to 2012 occurring among residents of California and Texas, the two most populous states, accounting for 47% of US Latinos. This cross-sectional analysis, based on granular data obtained from death certificates on cause of death, age, race, ethnicity and birthplace, makes use of normal standardization techniques and negative binomial regression models.

**Results:**

While Latinos overall have lower all-cancers-combined mortality rates than NLWs, these numbers were largely driven by low rates among the foreign born while mortality rates for US-born Latinos approach those of NLWs. Among Texas males, rates were 210 per 100,000 for NLWs and 166 for Latinos combined, but 201 per 100,000 for US-born Latinos and 125 for foreign-born Latinos. Compared to NLWs, US-born Latino males in California had mortality rate ratios of 2.83 (95% CI: 2.52–3.18) for liver cancer, 1.44 (95% CI: 1.30–1.61) for kidney cancer, and 1.25 (95% CI: 1.17–1.34) for colorectal cancer (CRC). Texas results showed a similar site-specific pattern.

**Conclusions:**

Specific cancer patterns for US-born Latinos, who have relatively high cancer mortality, similar overall to NLWs, are masked by aggregation of all Latinos, US-born and foreign-born. While NLWs had high mortality for lung cancer, US-born Latinos had high mortality for liver, kidney and male colorectal cancers. HCV testing and reinforcement of the need for CRC screening should be a priority in this specific and understudied population. The unprecedented proximity of overall rates between NLWs and US-born Latino populations runs counter to the prevailing narrative of Latinos having significantly lower cancer risk and mortality. Birthplace data are critical in detecting meaningful differences among Latinos; these findings merit not only clinical but also public health attention.

## Background

Cancer accounts for 22% of all deaths among Latinos in the United States (US), a population of 55 million [1, 2]. Yet, Latinos suffer a lower burden of cancer compared to both non-Latino black (NLB) and non-Latino white (NLW) populations, including lower incidence for almost all cancers except gall bladder and infection-related cancers: cervix, liver and stomach [1, 3]. Because overall incidence is lower among Latinos, overall cancer mortality tends to also be lower [1, 4]. These observed advantages may be partially due to the Healthy Immigrant Effect, whereby low incidence and mortality are the result of a steady immigrant influx of lower-risk populations [5, 6].

Acculturation, the complex process by which members of a foreign-born minority population adapt to traits from a prevailing majority [7], has been shown to change several important risk factors for cancer [8, 9], including increased prevalence of tobacco smoking, obesity, metabolic syndrome, diabetes, and hepatitis C virus infection with longer time spent in the US [9]. Consequently, the more acculturated US-born Latinos may be at higher risk for cancer.

Several studies that examined overall cancer mortality by birthplace found higher rates for US-born populations compared to their foreign-born counterparts [10–13]. Thus, aggregating cancer rates for any US minority population with significant immigrant proportions, whether Latino, Asian, or Black, may obscure important differences. Moreover, for the whole US, or within each state, the relative weight of the foreign-born population in each racial or ethnic group modifies that group’s observed cancer patterns. For example, while the foreign-born proportion of Blacks in the US is low overall, states such as Florida and New York, with relatively high proportions of Black Caribbean immigrants, have lower cancer mortality among Blacks than the US average [13].

For Latinos, detailed mortality analyses by cancer site according to birthplace are not available. We address this data gap and examine cancer mortality data from California (CA) and Texas (TX), the two states with the largest Latino populations in the US, 14 and 9.5 million respectively, comprising 47% of all US Latinos in 2010 [2]. By comparing cancer mortality in Latinos stratified by birthplace with NLWs and NLBs, we aim to provide a detailed description of cancer outcomes, particularly highlighting differences between two distinct Latino populations: those 36 million (65%) that are US-born, and the 19 million (35%) that are foreign-born [14]. This information will be valuable to health policy makers tasked with reducing disparities and monitoring the health outcomes of this burgeoning US minority population.

## Methods

Mortality data for 5 years, January 1, 2008 through December 31, 2012, were obtained from the California Department of Health Center for Health Statistics and Informatics and the Texas Department of State Health Services. Among the resident cases in each state, we analyzed 20 common causes of cancer deaths as well as all-sites-combined cancer which included all cases of malignant cancers. Cancer site was coded according to the International Statistical Classification of Diseases 10th revision. Ethnicity text fields and birthplace were examined in detail to obtain accurate race/ethnicity group information for each decedent, thereby minimizing misclassification. Population denominators for the states of California and Texas were obtained from the 5-year American Community Survey (2008–2012) [15].

Cancer mortality rates for 2008–2012 were calculated per 100,000 persons, by sex, annualized and age-standardized to the 2000 US Standard Population using 18 age group bands, all 5-year except the last, 85 and older. Corresponding 95% confidence intervals (CIs) for mortality rates were calculated with gamma intervals modification. To directly compare rates between Latinos in aggregate, US-born Latinos, foreign-born Latinos and the referent NLW population, we computed age-adjusted site-specific mortality rate ratios using negative binomial regression. Models included decedents ages 40 and over.

SAS 9.3 was used for data analysis. This study was approved by the University of Nevada, Las Vegas Institutional Review Board. Data use agreements were obtained from each state.

## Results

Cancer was the cause of death for 282,733 Californians and 183,018 Texans in 2008–2012. Among these, 44,283 (16%) in California and 33,073 (18%) in Texas were of Latino ethnicity. Of these Latino decedents, 43% in California and 33% in Texas were born outside of the 50 US states (Table 1).

**Table 1:** Characteristics of the Study Population by State, 2008–2012

	Population Data (Census 2010 and American Community Survey)	Cancer Mortality Data (2008–2012)		
	Total Population	Deaths from Cancer	% Cancer/All Deaths	% Foreign-born within racial/ethnic group
CALIFORNIA				
Non-Latino White	14,956,253	185,789	24%	10%
Non-Latino Black	2,436,082	21,024	23%	3%
Latino	14,013,719	44,283	23%	57%
US-Born	8,580,121	18,920	20%	0%
Foreign-Born	5,433,598	25,363	25%	100%
Mexican	12,055,090	35,832	22%	53%
Central American	1,193,268	3845	25%	96%
South American	309,569	1757	30%	96%
Caribbean^a^	305,901	1652	21%	77%
Other Latino^b^	149,891	1197	23%	15%
TEXAS				
Non-Latino White	11,397,345	122,899	22%	2%
Non-Latino Black	3,019,318	22,690	23%	1%
Latino	9,460,921	33,073	20%	33%
US-Born	6,458,501	22,034	19%	0%
Foreign-Born	3,002,420	11,039	25%	100%

^a^ Includes Caribbean Latinos (Dominican Republic, Cuba and Puerto Rico)

^b^ Includes those of Spaniard (European Spanish) origin or birthplace Spain

The leading causes of cancer mortality among Latinos overall were lung, prostate, female breast, colorectal (CRC), liver and pancreatic cancers, with only slight differences between the two states. Among all analyzed groups, foreign-born Latinos had the lowest all-cancers-combined mortality rates and NLBs had the highest. By cancer site, there was considerable heterogeneity: Latino mortality rates were lower than NLWs and NLBs for lung, breast and bladder cancers, among others; however, for stomach and cervical cancers, rates were similar to NLBs, and significantly higher than NLWs. For colorectal cancer, US-born Latino males in both states had high mortality rates, surpassed only by NLBs. In both states, liver and kidney mortality rates for US-born Latinos were the highest of all analyzed populations (Tables 2 and 3).

**Table 2:** Annual Age-Adjusted^a^ Mortality Rates for Selected Cancers per 100,000, California, 2008–2012

	All Combined^b^			Non-Latino White			Non-Latino Black			All Latino			Foreign-born Latino			US-born Latino		
	N	Rate	95% CI	N	Rate	95% CI	N	Rate	95% CI	N	Rate	95% CI	N	Rate	95% CI	N	Rate	95% CI
Male																		
Oral Cavity and Pharynx	3128	3.7	(3.6–3.9)	2088	4.2	(4.0–4.4)	207	4.3	(3.7–4.9)	400	2.5	(2.2–2.7)	217	2.1	(1.8–2.4)	183	3.1	(2.6–3.6)
Esophagus	4935	6.1	(6.0–6.3)	3705	7.6	(7.4–7.9)	207	4.8	(4.1–5.5)	667	4.4	(4.0–4.7)	349	3.5	(3.1–3.9)	318	5.7	(5.1–6.4)
Stomach	4474	5.6	(5.5–5.8)	1876	3.9	(3.8–4.1)	369	8.8	(7.9–9.8)	1316	8.2	(7.7–8.7)	909	8.7	(8.1–9.4)	407	7.3	(6.6–8.1)
Colorectum	13,249	16.8	(16.5–17.1)	8114	17.0	(16.6–17.4)	1115	26.1	(24.5–27.7)	2380	15.5	(14.9–16.2)	1237	12.7	(12.0–13.6)	1143	20.0	(18.8–21.2)
Liver	9076	10.7	(10.4–10.9)	4028	8.0	(7.7–8.2)	685	13.3	(12.3–14.4)	2360	14.0	(13.4–14.6)	1078	10.7	(10.0–11.4)	1282	19.8	(18.6–20.9)
Gallbladder	353	0.4	(0.4–0.5)	169	0.3	(0.3–0.4)	17	0.4	(0.2–0.6)	102	0.7	(0.6–0.9)	57	0.6	(0.5–0.9)	45	0.8	(0.6–1.1)
Pancreas	9483	11.9	(11.7–12.2)	6126	12.6	(12.3–12.9)	696	15.7	(14.4–16.9)	1598	10.7	(10.1–11.2)	929	10.1	(9.4–10.9)	669	11.7	(10.8–12.7)
Lung	34,113	44.3	(43.8–44.8)	23,578	49.5	(48.8–50.1)	2718	63.5	(61.0–66.1)	3586	26.6	(25.7–27.5)	2079	25.2	(24.0–26.4)	1507	29.0	(27.5–30.6)
Melanoma	3148	3.9	(3.8–4.1)	2887	6.1	(5.9–6.3)	22	0.4	(0.3–0.7)	186	1.1	(1.0–1.3)	127	1.3	(1.1–1.6)	59	0.9	(0.7–1.2)
Prostate	15,326	21.4	(21.1–21.8)	10,406	22.3	(21.9–22.7)	1696	48.6	(46.3–51.1)	2212	19.2	(18.4–20.1)	1291	18.3	(17.3–19.4)	921	20.8	(19.4–22.2)
Kidney	4170	5.2	(5.0–5.3)	2639	5.4	(5.2–5.7)	264	5.7	(5.0–6.5)	890	5.6	(5.2–6.0)	453	4.5	(4.1–5.0)	437	7.2	(6.5–8.0)
Bladder	5037	6.9	(6.7–7.1)	4018	8.5	(8.3–8.8)	221	5.8	(5.0–6.6)	472	3.8	(3.5–4.2)	260	3.5	(3.1–4.0)	212	4.3	(3.7–5.0)
Brain	4507	5.3	(5.2–5.5)	3201	6.8	(6.6–7.1)	171	3.4	(2.9–4.0)	777	3.7	(3.4–4.0)	445	3.8	(3.4–4.2)	332	3.6	(3.1–4.1)
CUP	6686	8.5	(8.3–8.7)	4571	9.5	(9.2–9.8)	464	10.6	(9.6–11.7)	1019	6.7	(6.3–7.2)	585	6.2	(5.6–6.8)	434	7.5	(6.8–8.3)
NHL	5817	7.5	(7.3–7.7)	3861	8.2	(8.0–8.5)	265	5.9	(5.2–6.7)	1109	7.2	(6.7–7.7)	693	7.4	(6.8–8.1)	416	7.1	(6.4–7.8)
Leukemia	6685	8.6	(8.4–8.8)	4512	9.8	(9.5–10.1)	357	8.4	(7.5–9.4)	1179	6.2	(5.8–6.6)	598	5.9	(5.3–6.5)	581	6.7	(6.1–7.5)
All-sites-combined	145,045	185.9	(185.0–186.9)	95,579	200.7	(199.4–202.0)	10,567	250.4	(245.4–255.6)	22,838	152.0	(149.8–154.1)	12,691	138.9	(136.2–141.6)	10,147	174.1	(170.5–177.8)
Female																		
Oral Cavity and Pharynx	1452	1.4	(1.4–1.5)	960	1.6	(1.5–1.7)	100	1.7	(1.4–2.1)	179	0.9	(0.8–1.1)	104	0.9	(0.7–1.1)	75	1.0	(0.8–1.3)
Esophagus	1422	1.4	(1.3–1.5)	1038	1.7	(1.6–1.8)	105	1.8	(1.4–2.1)	138	0.7	(0.6–0.9)	70	0.6	(0.5–0.8)	68	0.9	(0.7–1.2)
Stomach	3282	3.3	(3.2–3.4)	1171	1.9	(1.8–2.0)	282	5.0	(4.4–5.6)	1077	5.2	(4.8–5.5)	746	5.6	(5.2–6.0)	331	4.4	(3.9–4.9)
Colorectum	12,559	12.2	(12.0–12.4)	8024	12.9	(12.6–13.2)	1112	19.4	(18.2–20.6)	1803	9.2	(8.8–9.7)	1034	8.5	(7.9–9.0)	769	10.5	(9.8–11.3)
Liver	4469	4.5	(4.3–4.6)	1907	3.2	(3.0–3.3)	323	5.4	(4.8–6.0)	1220	6.5	(6.1–6.9)	736	6.3	(5.9–6.8)	484	6.8	(6.2–7.5)
Gallbladder	785	0.8	(0.7–0.8)	333	0.5	(0.5–0.6)	44	0.8	(0.6–1.0)	294	1.6	(1.4–1.7)	221	1.9	(1.6–2.2)	73	1.0	(0.8–1.3)
Pancreas	9550	9.5	(9.3–9.7)	6029	9.9	(9.6–10.2)	753	13.3	(12.4–14.3)	1606	8.6	(8.2–9.1)	976	8.5	(8.0–9.1)	630	8.9	(8.2–9.7)
Lung	30,575	30.9	(30.5–31.2)	22,888	38.7	(38.2–39.3)	2223	38.7	(37.1–40.4)	2512	13.6	(13.1–14.2)	1334	11.8	(11.1–12.4)	1178	16.8	(15.9–17.9)
Melanoma	1599	1.6	(1.5–1.7)	1395	2.5	(2.3–2.6)	16	0.3	(0.2–0.4)	137	0.7	(0.6–0.8)	93	0.7	(0.6–0.9)	44	0.6	(0.4–0.8)
Breast	21,696	21.5	(21.2–21.8)	14,177	24.6	(24.1–25.0)	1960	32.7	(31.2–34.2)	3335	15.1	(14.6–15.7)	1977	13.9	(13.2–14.5)	1358	17.3	(16.3–18.2)
Cervix	2207	2.2	(2.2–2.3)	959	1.9	(1.8–2.1)	218	3.6	(3.1–4.1)	718	2.9	(2.7–3.1)	481	3.1	(2.8–3.4)	237	2.5	(2.2–2.9)
Endometrium	4444	4.4	(4.3–4.5)	2683	4.5	(4.3–4.7)	479	8.1	(7.4–8.9)	773	3.7	(3.4–4.0)	464	3.4	(3.1–3.8)	309	4.1	(3.7–4.6)
Ovary	7758	7.7	(7.6–7.9)	5186	8.9	(8.6–9.1)	447	7.6	(6.9–8.4)	1317	6.3	(6.0–6.7)	810	6.1	(5.7–6.6)	507	6.7	(6.1–7.4)
Kidney	2304	2.3	(2.2–2.4)	1406	2.3	(2.2–2.4)	133	2.3	(2.0–2.8)	528	2.7	(2.4–2.9)	281	2.3	(2.1–2.7)	247	3.3	(2.9–3.7)
Bladder	2071	2.0	(1.9–2.1)	1532	2.3	(2.2–2.5)	161	2.9	(2.5–3.4)	233	1.3	(1.1–1.5)	126	1.2	(1.0–1.4)	107	1.5	(1.2–1.8)
Brain	3489	3.5	(3.4–3.6)	2378	4.4	(4.2–4.6)	158	2.7	(2.3–3.1)	649	2.8	(2.6–3.0)	376	2.9	(2.6–3.2)	273	2.6	(2.2–2.9)
CUP	6229	6.1	(6.0–6.3)	4248	7.0	(6.8–7.2)	477	8.2	(7.5–9.0)	932	4.8	(4.5–5.1)	542	4.5	(4.1–5.0)	390	5.3	(4.7–5.8)
NHL	4715	4.6	(4.5–4.8)	3026	4.8	(4.6–5.0)	221	3.9	(3.4–4.5)	916	4.8	(4.5–5.2)	529	4.5	(4.1–5.0)	387	5.3	(4.8–5.9)
Leukemia	5158	5.1	(5.0–5.3)	3328	5.5	(5.4–5.8)	304	5.3	(4.7–6.0)	956	4.1	(3.9–4.4)	58	4.2	(3.8–4.6)	448	4.2	(3.8–4.7)
All-sites-combined	137,688	136.9	(136.2–137.7)	90,209	151.8	(150.8–152.8)	10,457	180.1	(176.6–183.6)	21,445	106.4	(104.9–107.9)	12,672	101.6	(99.8–103.5)	8773	114.7	(112.2–117.2)

*Abbreviations*: *CUP* cancers of unknown primary, *NHL* non-Hodgkin lymphoma; All-sites-combined includes all cancers, not only those listed here

^a^ 2000 US Standard Population

^b^ Includes all race/ethnicities

**Table 3:** Annual Age-Adjusted^a^ Mortality Rates for Selected Cancers per 100,000, Texas, 2008–2012

	All Combined^b^			Non-Latino White			Non-Latino Black			All Latino			Foreign-born Latino			US-born Latino		
	N	Rate	95% CI	N	Rate	95% CI	N	Rate	95% CI	N	Rate	95% CI	N	Rate	95% CI	N	Rate	95% CI
Male																		
Oral Cavity and Pharynx	2108	4.0	(3.8–4.2)	1447	4.3	(4.1–4.5)	274	5.6	(4.9–6.4)	325	2.7	(2.4–3.0)	103	2.0	(1.6–2.5)	222	3.3	(2.9–3.8)
Esophagus	3134	6.1	(5.9–6.4)	2265	6.8	(6.5–7.1)	298	6.5	(5.7–7.4)	517	4.8	(4.4–5.3)	150	3.0	(2.5–3.6)	367	6.2	(5.6–6.9)
Stomach	2462	4.9	(4.7–5.1)	1052	3.3	(3.1–3.5)	403	9.4	(8.4–10.5)	878	7.8	(7.3–8.4)	304	6.1	(5.3–6.9)	574	9.3	(8.5–10.1)
Colorectum	9295	19.0	(18.6–19.4)	5698	18.1	(17.6–18.6)	1357	30.3	(28.5–32.1)	2009	18.4	(17.6–19.3)	556	11.6	(10.6–12.8)	1453	24.1	(22.8–25.5)
Liver	6153	11.4	(11.1–11.7)	2956	8.6	(8.3–9.0)	903	16.3	(15.2–17.5)	2004	17.1	(16.3–18.0)	503	10.5	(9.5–11.6)	1501	23.0	(21.7–24.2)
Gallbladder	240	0.5	(0.4–0.6)	118	0.4	(0.3–0.4)	29	0.8	(0.5–1.1)	79	0.8	(0.6–1.0)	29	0.6	(0.4–1.0)	50	0.9	(0.6–1.1)
Pancreas	5861	11.9	(11.6–12.2)	3902	12.1	(11.7–12.5)	670	15.2	(14.0–16.5)	1152	10.7	(10.1–11.4)	373	7.9	(7.0–8.8)	779	13.1	(12.1–14.1)
Lung	27,382	56.9	(56.2–57.6)	20,235	63.1	(62.2–64.0)	3492	81.9	(78.9–84.9)	3114	32.2	(31.0–33.4)	1044	25.2	(23.5–26.9)	2070	37.9	(36.2–39.6)
Melanoma	1824	3.7	(3.6–3.9)	1654	5.3	(5.0–5.5)	21	0.5	(0.3–0.8)	136	1.3	(1.0–1.5)	54	1.2	(0.9–1.6)	82	1.3	(1.0–1.7)
Prostate	8058	19.8	(19.3–20.2)	5280	18.7	(18.2–19.2)	1264	39.6	(37.3–42.0)	1405	17.6	(16.7–18.6)	557	16.3	(14.9–17.8)	848	18.6	(17.3–19.9)
Kidney	3286	6.6	(6.4–6.8)	2123	6.6	(6.3–6.9)	298	6.7	(5.8–7.5)	807	7.1	(6.6–7.6)	232	4.6	(4.0–5.4)	575	9.2	(8.4–10.0)
Bladder	2769	6.5	(6.2–6.7)	2251	7.7	(7.3–8.0)	190	5.3	(4.5–6.2)	279	3.1	(2.7–3.5)	91	2.2	(1.7–2.8)	188	3.8	(3.2–4.4)
Brain	2618	4.9	(4.7–5.1)	1921	6.0	(5.7–6.2)	162	3.0	(2.5–3.6)	476	3.4	(3.0–3.7)	178	2.9	(2.5–3.5)	298	3.7	(3.2–4.2)
CUP	5015	10.3	(10.0–10.6)	3421	10.8	(10.4–11.2)	558	12.4	(11.3–13.6)	917	8.3	(7.7–8.9)	289	6.5	(5.7–7.4)	628	9.9	(9.1–10.8)
NHL	3593	7.7	(7.5–8.0)	2489	8.2	(7.9–8.5)	264	5.7	(5.0–6.5)	768	7.1	(6.6–7.7)	249	5.3	(4.6–6.1)	519	8.6	(7.8–9.5)
Leukemia	4260	9.2	(8.9–9.5)	2950	9.9	(9.5–10.2)	384	8.9	(8.0–10.0)	832	6.9	(6.3–7.4)	248	5.4	(4.6–6.3)	584	8.3	(7.5–9.1)
All-sites-combined	97,730	203.3	(202.0–204.6)	66,085	210.1	(208.5–211.8)	11,736	274.7	(269.3–280.3)	17,668	166.4	(163.8–169.1)	5629	124.8	(121.2–128.5)	12,039	201.4	(197.6–205.3)
Female																		
Oral Cavity and Pharynx	837	1.3	(1.2–1.4)	607	1.5	(1.4–1.7)	97	1.5	(1.2–1.8)	108	0.8	(0.7–1.0)	31	0.5	(0.4–0.8)	77	1.0	(0.8–1.3)
Esophagus	726	1.2	(1.1–1.3)	518	1.3	(1.2–1.4)	88	1.4	(1.1–1.7)	109	0.9	(0.7–1.0)	36	0.7	(0.5–1.0)	73	1.0	(0.8–1.2)
Stomach	1728	2.8	(2.7–2.9)	679	1.7	(1.6–1.9)	262	4.3	(3.8–4.9)	700	4.9	(4.5–5.2)	262	3.9	(3.5–4.5)	438	5.6	(5.0–6.1)
Colorectum	7762	12.5	(12.2–12.8)	5025	12.6	(12.2–12.9)	1213	19.8	(18.6–20.9)	1342	9.9	(9.3–10.4)	379	6.3	(5.6–7.0)	963	12.6	(11.8–13.5)
Liver	2831	4.6	(4.5–4.8)	1374	3.5	(3.3–3.7)	322	5.0	(4.5–5.6)	996	7.8	(7.3–8.3)	347	6.2	(5.5–6.9)	649	9.0	(8.3–9.7)
Gallbladder	474	0.8	(0.7–0.8)	229	0.6	(0.5–0.7)	55	0.9	(0.7–1.2)	172	1.3	(1.1–1.5)	72	1.2	(0.9–1.6)	100	1.3	(1.1–1.6)
Pancreas	5505	9.0	(8.8–9.3)	3552	8.9	(8.6–9.2)	790	13.5	(12.6–14.5)	1031	8.0	(7.5–8.5)	359	6.5	(5.8–7.2)	672	9.2	(8.5–9.9)
Lung	20,606	33.9	(33.5–34.4)	16,047	41.0	(40.3–41.6)	2318	38.4	(36.8–40.0)	1806	14.0	(13.4–14.7)	677	12.2	(11.2–13.2)	1129	15.5	(14.6–16.5)
Melanoma	892	1.4	(1.3–1.5)	765	2.0	(1.9–2.2)	25	0.4	(0.3–0.6)	93	0.6	(0.5–0.8)	40	0.6	(0.4–0.8)	53	0.7	(0.5–0.9)
Breast	13,264	21.2	(20.8–21.5)	8205	21.1	(20.7–21.6)	2212	33.5	(32.1–35.0)	2518	16.5	(15.8–17.2)	931	13.4	(12.5–14.3)	1587	19.2	(18.3–20.2)
Cervix	1733	2.8	(2.6–2.9)	800	2.3	(2.2–2.5)	286	4.1	(3.7–4.7)	608	3.6	(3.3–3.9)	233	3.2	(2.8–3.7)	375	4.1	(3.7–4.5)
Endometrium	2307	3.7	(3.5–3.9)	1266	3.2	(3.0–3.4)	420	6.9	(6.2–7.6)	545	3.7	(3.4–4.0)	187	2.9	(2.5–3.4)	358	4.4	(4.0–4.9)
Ovary	4512	7.3	(7.1–7.5)	3092	7.9	(7.7–8.2)	425	7.0	(6.3–7.7)	880	6.1	(5.7–6.5)	304	4.7	(4.1–5.3)	576	7.2	(6.6–7.8)
Kidney	1834	3.0	(2.8–3.1)	1119	2.8	(2.6–3.0)	204	3.3	(2.9–3.8)	474	3.6	(3.2–3.9)	159	2.7	(2.3–3.2)	315	4.3	(3.8–4.8)
Bladder	1116	1.8	(1.7–2.0)	804	2.0	(1.8–2.1)	146	2.6	(2.2–3.0)	155	1.2	(1.0–1.4)	49	1.0	(0.7–1.3)	106	1.5	(1.2–1.8)
Brain	2096	3.3	(3.2–3.5)	1517	4.1	(3.9–4.3)	141	2.1	(1.8–2.5)	388	2.3	(2.1–2.6)	120	2.2	(1.6–2.9)	268	2.8	(2.5–3.2)
CUP	4169	6.8	(6.6–7.0)	2802	7.0	(6.8–7.3)	508	8.3	(7.6–9.1)	751	5.6	(5.2–6.0)	276	4.8	(4.2–5.4)	475	6.3	(5.7–6.9)
NHL	2831	4.7	(4.5–4.8)	1952	4.9	(4.7–5.1)	201	3.3	(2.8–3.8)	613	4.7	(4.3–5.1)	231	4.2	(3.6–4.8)	382	5.2	(4.6–5.7)
Leukemia	3218	5.3	(5.1–5.5)	2189	5.6	(5.4–5.9)	299	5.0	(4.4–5.6)	644	4.1	(3.7–4.4)	206	3.4	(2.9–4.0)	438	4.7	(4.2–5.2)
All-sites-combined	85,288	138.6	(137.6–139.5)	56,814	145.0	(143.8–146.2)	10,954	176.8	(173.4–180.2)	15,405	110.1	(108.3–111.9)	5410	89.0	(86.4–91.6)	9995	127.7	(125.1–130.3)

*Abbreviations*: *CUP* cancers of unknown primary, *NHL* non-Hodgkin lymphoma; All-sites-combined includes all cancers, not only those listed here

^a^ 2000 US Standard Population

^b^ Includes all race/ethnicities

Compared to NLWs, the risk of cancer death for Latinos in aggregate for all-cancers-combined was 23% and 26% lower in Texas and California, respectively, for both sexes combined (*p* < 0.05). However, in both states, significantly higher mortality was observed for stomach, cervix and gall bladder cancers for US-born and foreign-born Latinos compared to NLWs. Patterns for certain cancers, including liver, kidney and colorectal cancer, were markedly different between US-born and foreign-born Latinos, with significantly higher mortality seen in the US-born, consistent across both states. The largest difference was seen in liver cancer: US-born Latino men had 2.8 (95% CI: 2.5–3.2) and 2.7 (95% CI: 2.2–3.4) times higher liver cancer mortality than NLWs in California and Texas, respectively, while ratios for foreign-born Latino men were 1.2 (95%CI: 1.1–1.4) in California and 1.1 (95% CI: 0.9–1.3) in Texas (Table 4).

**Table 4:** Mortality Rate Ratios^a^ for Selected Cancers by Latino Ethnicity and Birthplace, CA and TX, 2008–2012

		California						Texas					
	Non-Latino White	All Latino		US-born Latino		Foreign-born Latino		All Latino		US-born Latino		Foreign-born Latino	
	Referent	RR	95% CI	RR	95% CI	RR	95% CI	RR	95% CI	RR	95% CI	RR	95% CI
Male													
Oral Cavity and Pharynx	1.00	0.57	(0.50–0.64)	0.74	(0.63–0.87)	0.46	(0.39–0.54)	0.62	(0.54–0.69)	0.79	(0.68–0.91)	0.42	(0.34–0.51)
Esophagus	1.00	0.56	(0.51–0.61)	0.74	(0.66–0.84)	0.45	(0.39–0.50)	0.65	(0.59–0.72)	0.86	(0.77–0.96)	0.41	(0.35–0.48)
Stomach	1.00	2.01	(1.86–2.18)	1.91	(1.66–2.18)	2.05	(1.82–2.30)	2.33	(2.13–2.55)	2.82	(2.55–3.13)	1.75	(1.54–1.99)
Colorectum	1.00	0.92	(0.88–0.97)	1.25	(1.17–1.34)	0.71	(0.66–0.76)	1.02	(0.96–1.07)	1.36	(1.28–1.45)	0.60	(0.55–0.66)
Liver	1.00	1.80	(1.69–1.92)	2.83	(2.52–3.18)	1.21	(1.07–1.37)	1.94	(1.68–2.25)	2.73	(2.22–3.36)	1.06	(0.85–1.32)
Gallbladder	1.00	2.14	(1.65–2.78)	2.59	(1.85–3.64)	1.83	(1.32–2.56)	2.07	(1.56–2.76)	2.40	(1.72–3.35)	1.67	(1.11–2.52)
Pancreas	1.00	0.83	(0.78–0.88)	0.99	(0.91–1.08)	0.73	(0.67–0.79)	0.87	(0.82–0.93)	1.09	(1.01–1.17)	0.62	(0.56–0.69)
Lung	1.00	0.48	(0.44–0.53)	0.57	(0.54–0.61)	0.44	(0.42–0.47)	0.46	(0.40–0.52)	0.56	(0.48–0.64)	0.34	(0.29–0.39)
Prostate	1.00	0.85	(0.81–0.89)	0.95	(0.88–1.02)	0.77	(0.73–0.83)	0.94	(0.88–1.00)	1.01	(0.92–1.09)	0.86	(0.78–0.95)
Kidney	1.00	1.06	(0.98–1.15)	1.44	(1.30–1.61)	0.82	(0.73–0.92)	1.09	(1.00–1.18)	1.45	(1.32–1.59)	0.67	(0.58–0.77)
Bladder	1.00	0.43	(0.38–0.47)	0.52	(0.44–0.60)	0.37	(0.32–0.42)	0.41	(0.36–0.46)	0.49	(0.42–0.57)	0.31	(0.25–0.38)
Brain	1.00	0.51	(0.46–0.56)	0.55	(0.48–0.64)	0.49	(0.43–0.55)	0.56	(0.50–0.62)	0.61	(0.53–0.70)	0.49	(0.42–0.58)
CUP	1.00	0.69	(0.64–0.75)	0.82	(0.74–0.92)	0.61	(0.55–0.67)	0.76	(0.71–0.82)	0.96	(0.88–1.05)	0.53	(0.47–0.60)
NHL	1.00	0.89	(0.82–0.96)	0.94	(0.84–1.04)	0.86	(0.78–0.94)	0.90	(0.83–0.98)	1.15	(1.00–1.31)	0.65	(0.56–0.76)
Leukemia	1.00	0.60	(0.56–0.66)	0.71	(0.63–0.81)	0.56	(0.49–0.63)	0.71	(0.65–0.79)	0.90	(0.79–1.02)	0.51	(0.44–0.60)
All-sites-combined	1.00	0.75	(0.58–0.96)	0.92	(0.90–0.94)	0.64	(0.63–0.65)	0.78	(0.76–0.80)	1.01	(0.93–1.09)	0.55	(0.51–0.60)
Female													
Oral Cavity and Pharynx	1.00	0.55	(0.46–0.67)	0.72	(0.56–0.91)	0.44	(0.34–0.57)	0.49	(0.40–0.61)	0.65	(0.51–0.83)	0.30	(0.20–0.43)
Esophagus	1.00	0.42	(0.34–0.51)	0.61	(0.47–0.79)	0.28	(0.20–0.38)	0.64	(0.52–0.78)	0.76	(0.59–0.97)	0.47	(0.33–0.66)
Stomach	1.00	2.52	(2.29–2.77)	2.37	(2.05–2.75)	2.65	(2.28–3.08)	2.86	(2.56–3.18)	3.24	(2.87–3.66)	2.37	(2.05–2.74)
Colorectum	1.00	0.70	(0.66–0.74)	0.85	(0.79–0.92)	0.58	(0.54–0.63)	0.77	(0.73–0.82)	1.00	(0.93–1.07)	0.49	(0.44–0.55)
Liver	1.00	2.04	(1.86–2.23)	2.25	(1.95–2.60)	1.81	(1.57–2.08)	1.98	(1.68–2.34)	2.36	(2.00–2.79)	1.51	(1.28–1.81)
Gallbladder	1.00	2.76	(2.33–3.28)	2.05	(1.58–2.66)	3.28	(2.70–3.98)	2.16	(1.77–2.64)	2.30	(1.82–2.91)	2.00	(1.52–2.60)
Pancreas	1.00	0.87	(0.82–0.92)	0.94	(0.86–1.02)	0.82	(0.76–0.89)	0.88	(0.82–0.94)	1.03	(0.94–1.11)	0.69	(0.62–0.77)
Lung	1.00	0.35	(0.32–0.39)	0.45	(0.40–0.50)	0.29	(0.26–0.33)	0.32	(0.28–0.37)	0.35	(0.31–0.41)	0.27	(0.23–0.32)
Breast	1.00	0.63	(0.60–0.66)	0.73	(0.68–0.78)	0.55	(0.52–0.59)	0.79	(0.75–0.83)	0.92	(0.87–0.98)	0.64	(0.59–0.68)
Cervix	1.00	1.51	(1.34–1.69)	1.45	(1.23–1.71)	1.61	(1.41–1.83)	1.57	(1.38–1.79)	1.81	(1.46–2.24)	1.41	(1.11–1.78)
Endometrium	1.00	0.88	(0.81–0.96)	1.00	(0.89–1.13)	0.79	(0.71–0.89)	1.20	(1.03–1.39)	1.44	(1.22–1.70)	0.90	(0.74–1.09)
Ovary	1.00	0.71	(0.67–0.76)	0.81	(0.73–0.89)	0.65	(0.59–0.71)	0.78	(0.72–0.84)	0.93	(0.85–1.02)	0.59	(0.53–0.67)
Kidney	1.00	1.29	(1.16–1.44)	1.60	(1.39–1.84)	1.07	(0.92–1.23)	1.27	(1.14–1.41)	1.52	(1.34–1.72)	0.95	(0.80–1.12)
Bladder	1.00	0.54	(0.46–0.64)	0.67	(0.54–0.82)	0.45	(0.36–0.56)	0.63	(0.53–0.74)	0.76	(0.62–0.93)	0.45	(0.34–0.61)
Brain	1.00	0.57	(0.51–0.63)	0.60	(0.51–0.71)	0.55	(0.48–0.63)	0.57	(0.50–0.64)	0.78	(0.63–0.91)	0.42	(0.32–0.55)
CUP	1.00	0.71	(0.66–0.77)	0.80	(0.72–0.90)	0.64	(0.58–0.72)	0.78	(0.72–0.85)	0.89	(0.81–0.93)	0.64	(0.56–0.72)
NHL	1.00	1.00	(0.92–1.09)	1.14	(1.02–1.28)	0.90	(0.80–1.00)	0.96	(0.87–1.05)	1.05	(0.94–1.17)	0.83	(0.73–0.96)
Leukemia	1.00	0.72	(0.65–0.81)	0.74	(0.64–0.86)	0.64	(0.56–0.73)	0.73	(0.64–0.83)	0.87	(0.74–1.03)	0.58	(0.48–0.70)
All-sites-combined	1.00	0.73	(0.58–0.92)	0.80	(0.78–0.82)	0.64	(0.63–0.66)	0.76	(0.74–0.77)	0.89	(0.86–0.91)	0.60	(0.58–0.62)

*Abbreviations*: *CUP* cancers of unknown primary, *NHL* non-Hodgkin lymphoma; All-sites-combined includes all cancers, not only those listed here

^a^ Negative binomial regression rate ratios adjusted for age groups 40+ years

## Discussion

This is the first detailed analysis of cancer mortality by site for Latinos disaggregated by birthplace, US-born vs foreign-born. While mortality rates for all Latinos combined were lower than for NLWs as expected, the profile changed substantially when birthplace was considered. Among US-born Latinos, males in Texas had similar overall mortality rates to NLWs (RR = 1.01; 95% CI 0.93–1.09), while Texas females were 11% lower; similarly, in California, mortality rates were 8% and 20% lower for males and females, respectively. This unprecedented proximity of overall rates between NLWs and US-born Latino populations runs counter to the prevailing narrative of Latinos having significantly better cancer outcomes [1, 16].

Theories of the negative effects of acculturation [8] might lead one to think that US-born Latino cancer mortality outcomes are simply converging with the majority NLW population. However, this is not uniformly the case; substantial heterogeneity was seen by cancer site. Non-Latino whites were more vulnerable to lung, breast, bladder and melanoma mortality, while US-born Latino mortality was excessive for liver, kidney and CRC (in males), as well as for stomach, cervix and gall bladder, previously documented [1]. Some of these results align with existing knowledge of racial/ethnic patterns in risk factors: for lung, breast, cervical, and stomach cancers, differences in prevalence of risk factors such as smoking, reproductive patterns, human papillomavirus (HPV) and *Helicobacter pylori* infection are explanatory [16, 17].

Additional results from this study are surprising, such as the similar or slightly higher rates for some cancers for US-born Latinos compared to NLWs. These include pancreas, endometrium, prostate cancer, and non-Hodgkin lymphoma (NHL), not previously shown to be this high in a predominantly Mexican Latino population. While not the sole risk factor, obesity is associated with increased risk of liver, kidney, CRC, pancreas and endometrial cancers [18]. Thus, the high prevalence of obesity documented among US-born Latinos [19, 20] suggests this should be a target for intervention.

Unique patterns deserving of further discussion include liver, kidney and CRC.

### Liver

The exceedingly high liver cancer mortality found in Latinos, especially among the US-born, whose rates are more than double those of NLWs, constitutes a true disparity. Unlike for NLWs, liver cancer was consistently one of the top four main causes of cancer death for both US-born and foreign-born male and female Latino populations. Our results confirm those from a previous mortality study using data through 2002 [21], as well as more recent incidence studies [22, 23].

Historically, liver cancer has been more common in developing countries and among US Latino and Asian immigrant populations, a pattern driven by their higher prevalence of hepatitis B infection (HBV) [24, 25]. With the implementation of HBV vaccination programs globally, this determinant of liver cancer, while still relevant, has reduced in prominence in the US [24, 26]. Instead, chronic infection with the hepatitis C virus (HCV) has been linked to the recent liver cancer incidence increases seen in the US, especially among the birth cohort of 1945–1965 [24, 26]. HCV infection in the US most often results from intravenous drug use and/or past transfusions with contaminated blood [26]. With the shifting roles of these two viral hepatitis infections, relative patterns for liver cancer between racial/ethnic groups in the US have also changed.

In our study, we found distinct patterns by gender. Foreign-born Latino men had liver cancer mortality rates similar to (California) or only slightly higher than (Texas) the referent NLW population. However, foreign-born Latina women in both states had significantly higher rates than NLW women, findings that are consistent with a recent study of diverse foreign-born Latinos in Florida [4]. Among US-born Latinos, liver cancer mortality rates were also higher for females compared to their NLW counterparts; however, they were exceedingly high for males, almost three times higher than NLWs in both states. While Latinos, especially the US-born, have high prevalence of some important risk factors for liver cancer [27], including obesity [9], diabetes [20], and heavy alcohol consumption among men [28], differences in HCV prevalence by gender and birthplace more likely explain the unique mortality patterns observed in this study.

Previous studies have attributed approximately 20% of US liver cancer cases to infection with HCV [27, 29]. However, these estimates are highly dependent upon methodology, especially the inclusion of relevant confounders. Bypassing these problems by using direct linkage between cancer registry data and viral hepatitis data, a recent study in New York City (NYC) found that a remarkable 40% of all NLW, 48% of all Latino, and 51% of all NLB new liver cancer cases in NYC were HCV-positive [30]. These results suggest that the role of HCV infection in the liver cancer “epidemic” may have been thus far underestimated. Additionally, regarding birthplace and HCV, researchers using NHANES data showed that US-born Latino males, with an elevated age-adjusted prevalence of HCV of 5.4%, have an approximately 8-times higher prevalence of HCV infection than their foreign-born Latino male counterparts [31]. Yet, among females, the prevalence ratio of HCV between US-born and foreign-born is comparatively lower, only 4-fold [31]. Furthermore, the overall prevalence of HCV among foreign-born Latinos was found to be lower than NLWs of both sexes [32]. Collectively, these data point towards the role of HCV prevalence in potentially explaining the differences in liver cancer mortality not only between US-born and foreign-born Latinos, but also between Latino males and females in relation to their NLW counterparts. Further research is needed to assess these gender-specific differences, especially given the likelihood that causal factors other than HCV play a larger role in liver cancer among the foreign-born, particularly among women. Moreover, this liver cancer disparity among US-born Latinos warrants specific interventions, possibly including targeted HCV screening and treatment as well as other public health measures aimed at reducing non-viral liver cancer risk factors in the Latino community, including obesity and metabolic disorders.

### Kidney

Mortality rates for kidney cancer were 44% higher in US-born Latino males than NLWs, and 52% (TX) and 60% (CA) higher in US-born females; foreign-born Latinos had lower (men) or similar (women) mortality from kidney cancer compared to NLWs. Obesity likely explains much of this disparity: the population-attributable fraction of overweight/obesity as a risk factor for kidney cancer has been estimated at over 40% [18]. US-born Latinos, especially US-born Mexicans, have a much higher prevalence of obesity than NLWs; historically foreign-born Latinos have had relatively lower prevalence of obesity, especially men [19, 20, 33]. Two additional known risk factors for kidney cancer are smoking and hypertension, the latter independent of obesity [34]. Yet, Latinos, even the US-born, smoke less than NLWs [20]. Notably, while hypertension prevalence is similar between Latinos and NLWs, treatment and control of hypertension is much lower in Latinos [20].

The high kidney cancer mortality rates found here in US-born Latinos approach national rates recorded among American Indians [35], previously documented with the highest kidney cancer burden in the US, for whom prevalence of obesity, smoking, and hypertension are universally high [16, 36]. These risk factors are common correlates of lower socio-economic status, a shared feature between American Indian and US-born Latinos. Both minority populations are disadvantaged in education level and poverty, as well as access to quality healthcare [20, 35]. The unique vulnerability of US-born Latino and American Indian populations to kidney cancer requires additional investigation and public health attention to fully understand and eliminate this disparity.

### Colorectal

In both states, US-born Latino men showed approximately 30% higher colorectal cancer mortality than NLW men, while mortality for US-born Latino women was only slightly lower (CA) or equivalent (TX) to their NLW counterparts. These findings contrast with previously recorded national rate ratios between Latinos in aggregate and NLWs during the same time period, 0.9 for men and 0.7 for women [1], demonstrating the importance of examining Latino cancer outcomes by birthplace. CRC risk factors that are high among US-born Latinos include obesity [9], diabetes [20], and heavy alcohol consumption among men [28], as previously mentioned. Additionally, low CRC screening among Latinos, especially men [20, 37], may further explain the disparity observed here. While other populations have seen declines in CRC mortality, attributed to increases in CRC screening [1], one recent study in California showed that low screening was driving a stable CRC mortality trend for Latinos [38]. Our findings suggest the same is happening in Texas; thus, this may be a problem with a national dimension. Given the high CRC mortality for US-born Latinos, continued efforts to increase the uptake of CRC screening and expand health care access are warranted in Latino communities.

This study presents valuable new data that provides evidence of cancer mortality disparities in the Latino population in the United States. Specific Latino ethnic group has been shown to be a major determinant of cancer mortality differences, as seen in Florida for Cubans, Puerto Ricans, Mexicans, Central and South Americans, and Dominicans [4]. However, independent of ethnic group, birthplace is a major determinant and confounder of cancer mortality rates, as shown here. Therefore, to generalize Latino cancer outcomes in the US without considering both ethnic group and birthplace is counterproductive. Across the US, regional variation in birthplace is remarkable, and confounds aggregate Latino rates by state and especially nationally. For example, in Florida an overwhelming 92% of Latino cancer decedents between 2008 and 2012 were foreign-born [4], while in this study only 43% were foreign-born in California and 33% in Texas. As an additional strength, our study benefits from very high completeness (>99%) of birthplace information for all decedents, which, combined with ethnicity and text descriptors, allowed for unprecedented reliable classification of Latinos by birthplace.

While it is possible that terminally ill Latinos could have returned to their home countries of origin to die, this out-migration has been found to be very small among Latinos [39, 40], and furthermore, would not affect the rates for US-born Latinos. Because we have previously shown Latino origin/ethnic group to be a major confounder in Latino cancer studies at large [4], we calculated disaggregated rates for Mexicans, Central Americans, and Other Latinos for the state of California, presented in Table 5. However, in California, 81% of all Latino decedents in our study were of Mexican origin, while Texas was even higher at 91%. Therefore, for these two states, not including origin/ethnic group data is unlikely to have biased our analysis.

**Table 5:** Annual Age-Adjusted^a^ Mortality Rates for Latino Ethnic Groups per 100,000, California, 2008–2012

	All Latino^a^			Mexican			Central American			South American			Caribbean		
	N	Rate	95% CI	N	Rate	95% CI	N	Rate	95% CI	N	Rate	95% CI	N	Rate	95% CI
MALE															
Oral Cavity and Pharynx	400	2.5	(2.2–2.7)	332	2.5	(2.2–2.8)	23	2.0	(1.1–3.1)	<10	1.5	(0.6–3.0)	22	3.7	(2.3–5.6)
Esophagus	667	4.4	(4.0–4.7)	558	4.4	(4.0–4.8)	40	4.0	(2.7–5.6)	14	2.6	(1.3–4.4)	31	5.4	(3.6–7.7)
Stomach	1316	8.2	(7.7–8.7)	1051	8.1	(7.6–8.6)	166	11.6	(9.5–14.0)	53	9.1	(6.7–12.0)	30	5.2	(3.5–7.5)
Colorectum	2380	15.5	(14.9–16.2)	1996	15.6	(14.9–16.4)	125	9.1	(7.3–11.3)	82	15.4	(12.1–19.4)	107	19.8	(16.1–23.9)
Liver	2360	14.0	(13.4–4.6)	2034	14.6	(13.9–15.3)	154	11.9	(9.8–14.4)	53	9.4	(6.9–12.5)	72	12.1	(9.4–15.3)
Gallbladder	102	0.7	(0.6–0.9)	86	0.7	(0.6–0.9)	<10	0.5	(0.2–1.2)	<10	1.1	(0.3–2.6)	<10	0.2	(0.0–1.0)
Pancreas	1598	10.7	(10.1–11.2)	1315	10.6	(10.0–11.2)	116	10.0	(8.0–12.4)	70	13.5	(10.3–17.2)	56	10.1	(7.5–13.1)
Lung	3586	26.6	(25.7–27.5)	2925	26.5	(25.4–27.5)	171	17.1	(14.3–20.2)	162	29.3	(24.7–34.4)	206	37.9	(32.8–43.5)
Melanoma	186	1.1	(1.0–1.3)	141	1.0	(0.9–1.2)	12	0.9	(0.4–1.7)	13	2.1	(1.1–3.7)	<10	1.4	(0.6–2.7)
Prostate	2212	19.2	(18.4–20.1)	1795	18.9	(18.1–19.9)	132	16.8	(13.8–20.2)	107	22.9	(18.6–27.9)	109	22.3	(18.3–26.9)
Kidney	890	5.6	(5.2–6.0)	754	5.7	(5.3–6.2)	44	2.7	(1.8–3.8)	30	5.1	(3.3–7.6)	38	4.5	(2.8–6.7)
Bladder	472	3.8	(3.5–4.2)	377	3.6	(3.2–4.0)	23	3.3	(2.0–4.9)	26	5.7	(3.7–8.2)	22	7.0	(4.9–9.6)
Brain	777	3.7	(3.4–4.0)	619	3.6	(3.3–3.9)	84	4.2	(3.2–5.6)	32	4.9	(3.3–7.1)	23	3.8	(2.4–5.8)
CUP	1019	6.7	(6.3–7.2)	838	6.6	(6.2–7.1)	69	5.5	(4.0–7.2)	44	8.3	(5.9–11.2)	39	6.8	(4.8–9.4)
NHL	1109	7.2	(6.7–7.7)	916	7.2	(6.7–7.7)	93	7.6	(5.9–9.7)	39	6.9	(4.8–9.6)	41	7.2	(5.1–9.8)
Leukemia	1179	6.2	(5.8–6.6)	970	6.0	(5.5–6.4)	105	6.6	(5.1–8.3)	42	7.3	(5.1–10.0)	32	5.8	(3.9–8.2)
All-sites-combined	22,838	152.0	(149.8–154.1)	18,863	151.6	(149.2–153.9)	1550	127.2	(119.6–135.1)	871	161.2	(150.1–173.0)	927	169.5	(158.6–181.0)
FEMALE															
Oral Cavity and Pharynx	179	0.9	(0.8–1.1)	136	0.9	(0.7–1.1)	23	1.2	(0.7–1.8)	<10	1.1	(0.5–2.1)	<10	0.9	(0.4–2.0)
Esophagus	138	0.7	(0.6–0.9)	104	0.7	(0.6–0.8)	17	0.9	(0.5–1.4)	<10	0.8	(0.3–1.7)	<10	0.7	(0.2–1.7)
Stomach	1077	5.2	(4.8–5.5)	805	4.9	(4.5–5.2)	193	8.2	(7.1–9.6)	47	5.7	(4.2–7.6)	10	1.4	(0.6–2.5)
Colorectum	1803	9.2	(8.8–9.7)	1376	8.9	(8.4–9.4)	188	8.8	(7.6–10.2)	89	10.9	(8.7–13.5)	89	12.2	(9.8–15.0)
Liver	1220	6.5	(6.1–6.9)	987	6.7	(6.3–7.2)	150	7.4	(6.2–8.7)	36	4.4	(3.1–6.2)	34	4.7	(3.3–6.6)
Gallbladder	294	1.6	(1.4–1.7)	227	1.5	(1.3–1.7)	44	2.3	(1.7–3.1)	12	1.5	(0.8–2.7)	<10	1.0	(0.4–2.1)
Pancreas	1606	8.6	(8.2–9.1)	1267	8.7	(8.2–9.2)	151	7.5	(6.3–8.8)	75	9.2	(7.2–11.6)	58	8.1	(6.1–10.5)
Lung	2512	13.6	(13.1–14.2)	1993	13.8	(13.2–14.4)	207	10.4	(8.9–11.9)	112	13.6	(11.2–16.4)	115	16.3	(13.4–19.5)
Melanoma	137	0.7	(0.6–0.8)	110	0.7	(0.5–0.8)	<10	0.3	(0.1–0.6)	<10	0.7	(0.2–1.5)	<10	0.7	(0.2–1.6)
Breast	3335	15.1	(14.6–15.7)	2672	15.1	(14.5–15.7)	317	12.7	(11.3–14.3)	133	15.2	(12.7–18.1)	129	18.1	(15.1–21.5)
Cervix	718	2.9	(2.7–3.1)	584	2.9	(2.7–3.2)	101	4.0	(3.2–5.0)	16	1.8	(1.0–2.9)	11	1.6	(0.7–2.8)
Endometrium	773	3.7	(3.4–4.0)	632	3.8	(3.5–4.1)	81	3.5	(2.8–4.4)	24	2.7	(1.7–4.1)	18	2.5	(1.5–3.9)
Ovary	1317	6.3	(6.0–6.7)	1033	6.3	(5.9–6.7)	172	7.3	(6.2–8.5)	47	5.3	(3.9–7.1)	36	5.0	(3.5–6.9)
Kidney	528	2.7	(2.4–2.9)	452	2.9	(2.6–3.2)	40	1.8	(1.2–2.4)	17	1.1	(0.5–2.1)	<10	1.2	(0.5–2.3)
Bladder	233	1.3	(1.1–1.5)	179	1.3	(1.1–1.5)	22	1.3	(0.8–1.9)	<10	2.1	(1.2–3.3)	<10	1.2	(0.5–2.3)
Brain	649	2.8	(2.6–3.0)	497	2.6	(2.4–2.9)	84	3.7	(2.9–4.6)	32	3.8	(2.6–5.4)	18	2.5	(1.5–4.0)
CUP	932	4.8	(4.5–5.1)	757	5.0	(4.6–5.4)	77	3.5	(2.7–4.4)	44	5.4	(3.9–7.2)	34	4.7	(3.2–6.5)
NHL	916	4.8	(4.5–5.2)	713	4.8	(4.4–5.1)	101	4.9	(4.0–6.1)	39	4.7	(3.3–6.4)	29	4.0	(2.7–5.8)
Leukemia	956	4.1	(3.9–4.4)	768	4.0	(3.7–4.3)	105	4.5	(3.6–5.5)	29	3.6	(2.4–5.3)	36	4.9	(3.4–6.8)
All-sites-combined	21,445	106.4	(104.9–107.9)	16,969	106.0	(104.4–107.7)	2295	104.0	(99.5–108.5)	886	106.8	(99.8–114.1)	725	101.6	(94.3–109.3)

Central American (major group, Salvadorans, 48%); South American (major group, Peruvians, 28%); Caribbean includes Puerto Ricans, Cubans, Dominicans

*Abbreviations*: *CUP* cancers of unknown primary, *NHL* non-Hodgkin lymphoma; All-sites-combined includes all cancers, not only those listed here

^a^ 2000 US Standard Population

^b^ Includes those of Spaniard (European Spanish) origin or birthplace Spain

Mortality is primarily a function of cancer incidence; however, it is possible that limited quality health care access for Latinos results in poor cancer survival, thus impacting the mortality burden. While analyzing any differential survival between US-born and foreign-born Latinos can be problematic [41, 42], the Surveillance, Epidemiology and End Results (SEER) program, which conducts follow-up for more than 95% of all cancer patients, shows almost no differences in overall survival between NLWs and its Latino population, overwhelmingly Mexican [43]. Therefore, the mortality differences seen in our study are likely driven by differences in cancer incidence, rather than survival. Notwithstanding, future accurate survival studies with complete follow-up, especially in Texas, a non-SEER state, are warranted to assess the contribution of differential survival to these mortality patterns.

## Conclusions

There are two main conclusions to be drawn from this study. First, in order for cancer indicators for Latinos to be accurate and useful in cancer prevention and control efforts, both ethnic group and birthplace must be taken into consideration. The Latino “advantage” in mortality does apply to foreign-born Latinos, but less so to US-born Latinos, suggesting that in aggregate, foreign-born status is the advantage, rather than ethnicity per se. Moreover, as previously suggested, the cancer advantage for Latinos seems, at least for men, to be largely an effect of tobacco smoking trends [19, 44]. If the excess in mortality from lung cancer (and melanoma whose rate is inherently higher) among NLWs were subtracted, overall mortality rates among US-born Latino men would be higher than NLWs in Texas, and similar in California.

Secondly, but more importantly, this elevated cancer mortality among US-born Latinos is an important and worrisome indicator. Since 2000, the share of foreign-born among Latinos has been declining, and birth has replaced migration as the primary source of population growth [14]. The role of negative acculturation among Latinos should be further studied given that the prevalence of many factors implicated in increased cancer mortality - HCV, obesity, diabetes, and uncontrolled hypertension – are now higher among US-born Latinos than NLWs, the host population to which Latinos supposedly acculturate. Already, current data from the California Health Information Survey shows only minimal differences between US-born and foreign born Latinos for smoking, obesity, and diabetes [45]. Thus, the offsetting of cancer rates by the “healthier” foreign-born among Latinos will not be a long-lasting trend. As US-born Latino rates for some cancers begin to approximate American Indian and African American populations, long known to be disadvantaged, the narrative describing Latino cancer outcomes will need to align with critical examination of the role of social determinants of health among US minority populations.

## Abbreviations

CA: California
CI: Confidence intervals
CRC: Colorectal cancer
CUP: Cancers of unknown primary
HBV: Hepatitis B virus
HCV: Hepatitis C virus
HPV: Human papillomavirus
NHL: Non-Hodgkin lymphoma
NLB: Non-Latino black
NLW: Non-Latino white
NYC: New York City
SEER: Surveillance, Epidemiology and End Results
TX: Texas
US: United States

## References

[1] Siegel RL, Fedewa SA, Miller KD, Goding-Sauer A, Pinheiro PS, Martinez-Tyson D (2015). Cancer statistics for Hispanics/Latinos, 2015. CA Cancer J Clin.

[2] U.S. Census Bureau, Census 2010: Summary File 1, Detailed Tables, Tables QT-PCT-10; Summary File 2, Detailed Tables, PCT-3. Available at https://factfinder.census.gov/faces/tableservices/jsf/pages/productview.xhtml?src=bkmk. Accessed 1 Oct 2016.

[3] Pinheiro PS, Sherman RL, Trapido EJ, Fleming LE, Huang Y, Gomez-Marin O (2009). Cancer incidence in first generation U.S. Hispanics: Cubans, Mexicans, Puerto Ricans, and new Latinos. Cancer Epidemiol Biomark Prev.

[4] Pinheiro PS, Callahan KE, Siegel RL, Jin H, Morris CR, Trapido EJ (2017). Cancer mortality in Hispanic ethnic groups. Cancer Epidemiol Biomark Prev.

[5] Kennedy S, Kidd MP, McDonald JT, Biddle N (2015). The healthy immigrant effect: patterns and evidence from four countries. J Int Migr Integr.

[6] Singh GK, Rodriguez-Lainz A, Kogan MD (2013). Immigrant health inequalities in the United States: use of eight major national data systems. ScientificWorldJournal.

[7] Schwartz SJ, Unger JB, Zamboanga BL, Szapocznik J (2010). Rethinking the concept of acculturation: implications for theory and research. Am Psychol.

[8] Abraido-Lanza AF, Chao MT, Florez KR (2005). Do healthy behaviors decline with greater acculturation?: implications for the Latino mortality paradox. Soc Sci Med.

[9] Lara M, Gamboa C, Kahramanian MI, Morales LS, Hayes Bautista DE (2005). Acculturation and Latino health in the United States: a review of the literature and its sociopolitical context. Annu Rev Public Health.

[10] Singh GK, Hiatt RA (2006). Trends and disparities in socioeconomic and behavioural characteristics, life expectancy, and cause-specific mortality of native-born and foreign-born populations in the United States, 1979–2003. Int J Epidemiol.

[11] Tao L, Ladabaum U, Gomez SL, Cheng I (2014). Colorectal cancer mortality among Hispanics in California: differences by neighborhood socioeconomic status and birthplace. Cancer.

[12] Eschbach K, Stimpson JP, Kuo Y, Goodwin JS (2007). Mortality of foreign-born and US-born Hispanic adults at younger ages: a reexamination of recent patterns. Am J Public Health.

[13] Pinheiro PS, Callahan K, Ragin C, Hage R, Hylton T, Kobetz E (2016). Black heterogeneity in cancer mortality: US-blacks, Haitians, and Jamaicans. Cancer Control.

[14] Stepler R, Brown A. Statistical Portrait of Hispanics in the United States. Pew Hispanic Center; April 19, 2016. Available at http://www.pewhispanic.org/2016/04/19/statistical-portrait-of-hispanics-in-the-unitedstates/.

[15] Ruggles S, Genadek KE, Goeken R, Grover J, and Sobek M. Integrated public use microdata series: version 6.0 [machine-readable database]. American community survey 2012 5-year estimates. Minneapolis: University of Minnesota. Accessed Sept 2016.

[16] Siegel RL, Miller KD, Jemal A (2016). Cancer statistics, 2016. CA Cancer J Clin.

[17] Howe HL, Wu X, Ries LA, Cokkinides V, Ahmed F, Jemal A (2006). Annual report to the nation on the status of cancer, 1975–2003, featuring cancer among US Hispanic/Latino populations. Cancer.

[18] Calle EE, Kaaks R (2004). Overweight, obesity and cancer: epidemiological evidence and proposed mechanisms. Nat Rev Cancer.

[19] Goldman N (2016). Will the Latino mortality advantage endure?. Res Aging.

[20] Dominguez K, Penman-Aguilar A, Chang M, Moonesinghe R, Castellanos T, Rodriguez-Lainz A (2015). Vital signs: leading causes of death, prevalence of diseases and risk factors, and use of health services among Hispanics in the United States—2009–2013. MMWR Morb Mortal Wkly Rep.

[21] HB E-S, Rudolph KL (2007). Hepatocellular carcinoma: epidemiology and molecular carcinogenesis. Gastroenterology.

[22] Setiawan VW, Wei PC, Hernandez BY, Lu SC, Monroe KR, Le Marchand L (2016). Disparity in liver cancer incidence and chronic disease mortality by birthplace in Hispanic : the multiethnic cohort. Cancer.

[23] Chang ET, Yang J, Alfaro-Velcamp T, So SK, Glaser SL, Gomez SL (2010). Disparities in liver cancer incidence by birthplace, acculturation, and socioeconomic status in California Hispanics and Asians. Cancer Epidemiol Biomark Prev.

[24] Ryerson AB, Eheman CR, Altekruse SF, Ward JW, Jemal A, Sherman RL (2016). Annual report to the nation on the status of cancer, 1975–2012, featuring the increasing incidence of liver cancer. Cancer.

[25] Schweitzer A, Horn J, Mikolajczyk RT, Krause G, Ott JJ (2015). Estimations of worldwide prevalence of chronic hepatitis B virus infection: a systematic review of data published between 1965 and 2013. Lancet.

[26] El-Serag HB (2012). Epidemiology of viral hepatitis and hepatocellular carcinoma. Gastroenterology.

[27] Makarova-Rusher OV, Altekruse SF, McNeel TS, Ulahannan S, Duffy AG, Graubard BI (2016). Population attributable fractions of risk factors for hepatocellular carcinoma in the United States. Cancer.

[28] Vaeth PA, Caetano R, Rodriguez LA (2012). The Hispanic Americans baseline alcohol survey (HABLAS): the association between acculturation, birthplace and alcohol consumption across Hispanic national groups. Addict Behav.

[29] Welzel TM, Graubard BI, Quraishi S, Zeuzem S, Davila JA, El-Serag HB (2013). Population-attributable fractions of risk factors for hepatocellular carcinoma in the United States. Am J Gastroenterol.

[30] Moore MS, Ivanina E, Bornschlegel K, Qiao B, Schymura MJ, Laraque F. Hepatocellular Carcinoma and Viral Hepatitis in New York City. Clinical Infectious Diseases. 2016;63(12):1577–83. 10.1093/cid/ciw605 27585801

[31] Kuniholm MH, Jung M, Everhart JE, Cotler S, Heiss G, McQuillan G (2014). Prevalence of hepatitis C virus infection in US Hispanic/Latino adults: results from the NHANES 2007–2010 and HCHS/SOL studies. J Infect Dis.

[32] Ditah I, Ditah F, Devaki P, Ewelukwa O, Ditah C, Njei B (2014). The changing epidemiology of hepatitis C virus infection in the United States: National Health and nutrition examination survey 2001 through 2010. J Hepatol.

[33] Singh GK, Siahpush M, Hiatt RA, Timsina LR (2011). Dramatic increases in obesity and overweight prevalence and body mass index among ethnic-immigrant and social class groups in the United States, 1976–2008. J Community Health.

[34] Chow W, Dong LM, Devesa SS (2010). Epidemiology and risk factors for kidney cancer. Nat Rev Urol.

[35] Li J, Weir HK, Jim MA, King SM, Wilson R, Master VA. Kidney cancer incidence and mortality among American Indians and Alaska Natives in the United States, 1990–2009. Am J Public Health. 2014;104(S3):S396–403. 10.2105/AJPH.2013.301616 PMC4035892 24754655

[36] Espey DK, Wu X, Swan J, Wiggins C, Jim MA, Ward E (2007). Annual report to the nation on the status of cancer, 1975–2004, featuring cancer in American Indians and Alaska natives. Cancer.

[37] Wallace SP, Gutiérrez VF, Castañeda X (2008). Access to preventive services for adults of Mexican origin. J Immigr Minor Health.

[38] Martinsen RP, Morris CR, Pinheiro PS, Parikh-Patel A, Kizer KW. Colorectal Cancer Trends in California and the Need for Greater Screening of Hispanic Men. Am J Prev Med. 2016;51(6):e155–e163. 10.1016/j.amepre.2016.05.019 27476382

[39] Abraido-Lanza AF, Dohrenwend BP, Ng-Mak DS, Turner JB (1999). The Latino mortality paradox: a test of the “salmon bias” and healthy migrant hypotheses. Am J Public Health.

[40] Turra CM, Elo IT (2008). The impact of salmon bias on the Hispanic mortality advantage: new evidence from social security data. Popul Res Policy Rev.

[41] Pinheiro PS (2013). The influence of Hispanic ethnicity on nonsmall cell lung cancer histology and patient survival. Cancer.

[42] Pinheiro PS, Morris CR, Liu L, Bungum TJ, Altekruse SF (2014). The impact of follow-up type and missed deaths on population-based cancer survival studies for Hispanics and Asians. J Natl Cancer Inst Monogr..

[43] Mariotto AB, Noone AM, Howlader N (2014). Cancer survival: an overview of measures, uses, and interpretation. J Natl Cancer Inst Monogr.

[44] Fenelon A (2013). Revisiting the Hispanic mortality advantage in the United States: the role of smoking. Soc Sci Med.

[45] UCLA Center for Health Policy Research. AskCHIS. Available at http://ask.chis.ucla.edu. Accessed 1 Aug 2016.

